# Adapting to Adulthood: A Review of Transition Strategies for Osteogenesis Imperfecta

**DOI:** 10.1007/s00223-024-01305-1

**Published:** 2024-11-13

**Authors:** Luca Celli, Mark R. Garrelfs, Ralph J. B. Sakkers, Mariet W. Elting, Mauro Celli, Arend Bökenkamp, Cas Smits, Thadé Goderie, Jan Maerten Smit, Lothar A. Schwarte, Patrick R. Schober, Wouter D. Lubbers, Marieke C. Visser, Arthur J. Kievit, Barend J. van Royen, Marjolijn Gilijamse, Willem H. Schreuder, Thomas Rustemeyer, Angela Pramana, Jan-Jaap Hendrickx, Max R. Dahele, Peerooz Saeed, Annette C. Moll, Katie R. Curro–Tafili, Ebba A. E. Ghyczy, Chris Dickhoff, Robert A. de Leeuw, Jaap H. Bonjer, Jakko A. Nieuwenhuijzen, Thelma C. Konings, Anton F. Engelsman, Augustinus M. Eeckhout, Joost G. van den Aardweg, Patrick J. Thoral, David P. Noske, Leander Dubois, Berend P. Teunissen, Oliver Semler, Lena Lande Wekre, Katre Maasalu, Aare Märtson, Luca Sangiorgi, Paolo Versacci, Mara Riminucci, Paola Grammatico, Anna Zambrano, Lorena Martini, Marco Castori, Esmee Botman, Ingunn Westerheim, Lidiia Zhytnik, Dimitra Micha, Elisabeth Marelise W. Eekhoff

**Affiliations:** 1 Department of Human Genetics, Amsterdam UMC Location VUmc, Amsterdam, The Netherlands; 2 Rare Bone Disease Center Amsterdam, Amsterdam, The Netherlands; 3 Amsterdam Reproduction and Development Research Institute, Amsterdam, The Netherlands; 4 Department of Endocrinology and Metabolism, Amsterdam UMC Location Vrije Universiteit, Amsterdam, The Netherlands; 5 Department of Experimental Medicine, Sapienza University of Rome, Rome, Italy; 6 Rare Bone Diseases Center, AOU Policlinico Umberto I, Sapienza University of Rome, Rome, Italy; 7 Department of Materna Infantile and Urological Sciences, Sapienza University of Rome, Rome, Italy; 8 Department of Pediatric Endocrinology, Amsterdam UMC, University of Amsterdam and Vrije Universiteit, Amsterdam, The Netherlands; 9 Amsterdam Gastroenterology Endocrinology Metabolism, Amsterdam, The Netherlands; 10 Emma Children’s Hospital, Amsterdam UMC, University of Amsterdam, Amsterdam, The Netherlands; 11 Department of Orthopedic Surgery, University Medical Center Utrecht, Utrecht, The Netherlands; 12 Department of Pediatric Nephrology, Emma Children’s Hospital, Amsterdam, The Netherlands; 13 Otolaryngology-Head and Neck Surgery, Section Ear and Hearing, Amsterdam UMC, Amsterdam, The Netherlands; 14 Amsterdam Public Health Research Institute, Amsterdam UMC, Amsterdam, The Netherlands; 15 Department of Plastic, Reconstructive- and Hand Surgery, Amsterdam UMC, Location VUmc, Amsterdam, The Netherlands; 16 Amsterdam Bone Centre, Amsterdam, The Netherlands; 17 Department of Anesthesiology, Amsterdam UMC, Amsterdam, The Netherlands; 18 Department of Neurology, Amsterdam University Medical Centre, Location AMC, Amsterdam, The Netherlands; 19 Department of Orthopedic Surgery and Sports Medicine, Amsterdam UMC Location AMC, Amsterdam, The Netherlands; 20 Amsterdam Movement Sciences Research Institute, Amsterdam, The Netherlands; 21 Department of Oral and Maxillofacial Surgery, Amsterdam UMC Location VUmc, Amsterdam, The Netherlands; 22 Dermato-Allergology and Occupational Dermatology, Amsterdam UMC, Amsterdam, The Netherlands; 23 Center for Special Care in Dentistry, Department of Maxillofacial Prosthodontics, Stichting Bijzondere Tandheelkunde, Amsterdam, The Netherlands; 24 Department of Otolaryngolgy/Head and Neck Surgery, Amsterdam UMC Location VUMC, Amsterdam, The Netherlands; 25 Cancer Center Amsterdam, Amsterdam, The Netherlands; 26 Department of Radiation Oncology, Amsterdam UMC, Location VUMC, Amsterdam, The Netherlands; 27 Orbital center, Department of ophthalmology, Amsterdam UMC, Amsterdam, The Netherlands; 28 Department of Ophthalmology, Amsterdam UMC, Location VUmc, Amsterdam, The Netherlands; 29 Department of Cardiothoracic Surgery, Amsterdam UMC Location Vrije Universiteit Amsterdam, Amsterdam, The Netherlands; 30 Department of Obstetrics and Gynecology, Amsterdam UMC, Location AMC, Amsterdam, The Netherlands; 31 Department of Surgery, Amsterdam University Medical Centre, Amsterdam, The Netherlands; 32 Department of Urology, Amsterdam UMC Location VUMC, Amsterdam, The Netherlands; 33 Department of Cardiology, Amsterdam UMC, Location University of Amsterdam, Amsterdam, Netherlands; 34 Department of Psychiatry, Amsterdam UMC, Amsterdam, The Netherlands; 35 Department of Respiratory Medicine, Amsterdam UMC, Location AMC, Amsterdam, The Netherlands; 36 Department of Intensive Care Medicine, Center for Critical Care Computational Intelligence, Amsterdam UMC, Vrije Universiteit, Amsterdam, The Netherlands; 37 Department of Neurosurgery, Amsterdam UMC Location VUmc, Amsterdam, The Netherlands; 38 Department of Radiology and Nuclear Medicine, Amsterdam UMC Location VUmc, Amsterdam, The Netherlands; 39 Department of Pediatrics, Faculty of Medicine and University Hospital Cologne, University of Cologne, Cologne, Germany; 40 TRS National Resource Center for Rare Disorders, Sunnaas Rehabilitation Hospital, Oslo, Norway; 41 Department of Orthopaedics, University of Tartu, Tartu, Estonia; 42 Clinic of Orthopaedics, Tartu University Hospital, Tartu, Estonia; 43 Department of Rare Skeletal Disorders, IRCCS Istituto Ortopedico Rizzoli, Bologna, Italy; 44 Department of Molecular Medicine, Sapienza University of Rome, Rome, Italy; 45 Department of Orthopaedic and Traumatology, “Sapienza” University of Rome, Rome, Italy; 46 Division of Medical Genetics, Fondazione IRCCS-Casa Sollievo Della Sofferenza, San Giovanni Rotondo, Foggia, Italy; 47 Osteogenesis Imperfecta Federation Europe (OIFE), Heffen, Belgium

**Keywords:** Osteogenesis imperfecta, Transitional care, Continuity of patient care, Adult care, Multidisciplinary care

## Abstract

Osteogenesis Imperfecta (OI), known as “brittle bone disease,” presents a rare genetic disorder characterized by bone fragility, often accompanied by skeletal deformities and extraskeletal complications. OI is primarily associated with collagen type I defects, responsible for the syndromic nature of the disease affecting a broad range of tissues. As such, its multisystemic complexity necessitates multidisciplinary care approaches in all patient life stages. OI treatment remains largely supportive, commonly including bisphosphonates and orthopedic surgeries, which show promise in children. Although rehabilitation programs for children exist, guidelines for adult care and especially the transition from pediatric to adult care, are lagging behind in OI care and research. The current systematic review summarizes the literature on OI patient pediatric to adult care transition experiences and compares OI transition approaches to other chronic diseases. The review was performed based on the Preferred Reporting Items for Systematic Reviews and Meta-Analyses (PRISMA) guidelines. Systematic searches were conducted across multiple databases. Search terms encompassed synonyms and closely related phrases relevant to “OI” and “Transition to adult care”. The initial screening involved the evaluation of article titles, followed by a thorough review of abstracts to assess relevance for the purpose of the current review. Programs aimed at easing the transition from pediatric to adult OI care necessitate a multifaceted approach. Collaborative efforts between different medical disciplines including pediatricians, endocrinologists, orthopedics, cardiology, pulmonology, ophthalmology, otolaryngologists, maxillofacial specialists, psychologists and medical genetics, are crucial for addressing the diverse needs of OI patients during this critical life phase. Comprehensive education, readiness assessments, personalized transition plans, and further follow-up are essential components of a structured transition framework. Further research is warranted to evaluate the feasibility and efficacy of sequential stepwise transition systems tailored to individuals with OI.

## Introduction

Osteogenesis Imperfecta (OI), also known as “brittle bone disease,” is a rare genetic disorder characterized by increased bone fragility which can result in hundreds of fractures during a person’s lifetime. In many cases, these are combined with severe skeletal deformities and short height, ligament laxity, dentinogenesis imperfecta, hearing loss and blue sclera [1]. OI is primarily associated with pathogenic variants in genes involved in the biosynthesis, post-translational modification or processing of type I collagen [2]. This is the prevalent protein component of the extracellular matrix in bones, but it is also abundant in many other connective tissues. Therefore, the affected collagen can compromise the function of multiple extraskeletal organ systems to various degrees [3–8]. Although most attention has focused on the skeletal involvement in OI, especially during childhood, extraskeletal manifestations appear to play a major role in increased risk of premature death in adulthood [7], which is mainly attributed to respiratory, cardiovascular and gastrointestinal complications [9]. The challenging multisystemic nature of OI necessitates multidisciplinary care approaches for the design of personalized treatment regimens which can deliver maximal benefit to the patient to counteract disease factors undermining quality of life [2, 3, 9–11].

The severity of OI can vary widely, with some individuals experiencing milder symptoms and others facing more severe challenges, which are partly related to the underlying qualitative or quantitative defects in collagen type I [12–15]. Based on the skeletal phenotype, OI patients are currently classified into five different clinical types according to the Sillence classification system [14, 16]. The vast majority of OI cases are caused by pathogenic variants in *COL1A1* and *COL1A2* genes, whereas the remaining ~ 10–15% of cases are associated with pathogenic variants in one of the more than 20 OI-causative genes; OI types 1 to 4 are genetically very heterogeneous being caused by an increasing number of pathogenic variants in numerous OI genes whereas OI type 5 is caused by one variant in the *IFITM5* gene [2, 17]. Although there is yet no targeted therapy for OI, bisphosphonates can have a positive effect on bone mineral density in children [18].

It is established that OI patients experience differently the impact of diverse OI symptoms on their quality of life depending on whether they are in the pediatric, adolescence or adult stage of life [19]. The complexity and evolving nature of OI challenges complicates transition programs for which patients report several issues including lack of clarity and adequate support [20, 21]. Healthcare providers currently struggle with limited guidelines providing clarity about best transition practice [22, 23]. Due to the high incidence of fractures in childhood and the associated disabilities during growth, rehabilitation programs for OI children were one of the first to be developed [24]. The follow-up guidelines for skeletal and extraskeletal manifestations in adulthood have only recently started receiving attention. However, advances in understanding of the natural disease history have allowed the realization of the first recommendations for follow-up care in childhood and adolescence [25–30]. Although studies about transition from pediatric to adult care in OI still remain scarce [31] lessons may be learned from transition programs which are developed for other chronic diseases. This transition phase is crucial to keep patients in care and to guide them to a different care approach which is compatible with their new phase of life. In this article, we summarize the existing literature on transition of care in OI, incorporating insights from other chronic diseases.

## Methods

### Literature Search

The analysis of literature for the review was conducted based on the Preferred Reporting Items for Systematic Reviews and Meta-Analyses (PRISMA) guidelines. In order to detect all publications relevant for the topic of the review, systematic searches in the bibliographic databases of PubMed, Web of Science (Core Collection), Scopus and Embase have been conducted from inception to April 2024.

The used search terms included (comprising synonyms and closely related terms) as index or free-text words: “Osteogenesis Imperfecta” AND “Continuity of Patient Care” OR “Transition to Adult Care” OR “Transitional Care”. The primary search did not include filtering for date or language of the publication.

### Selection Procedure

**Fig. 1:**
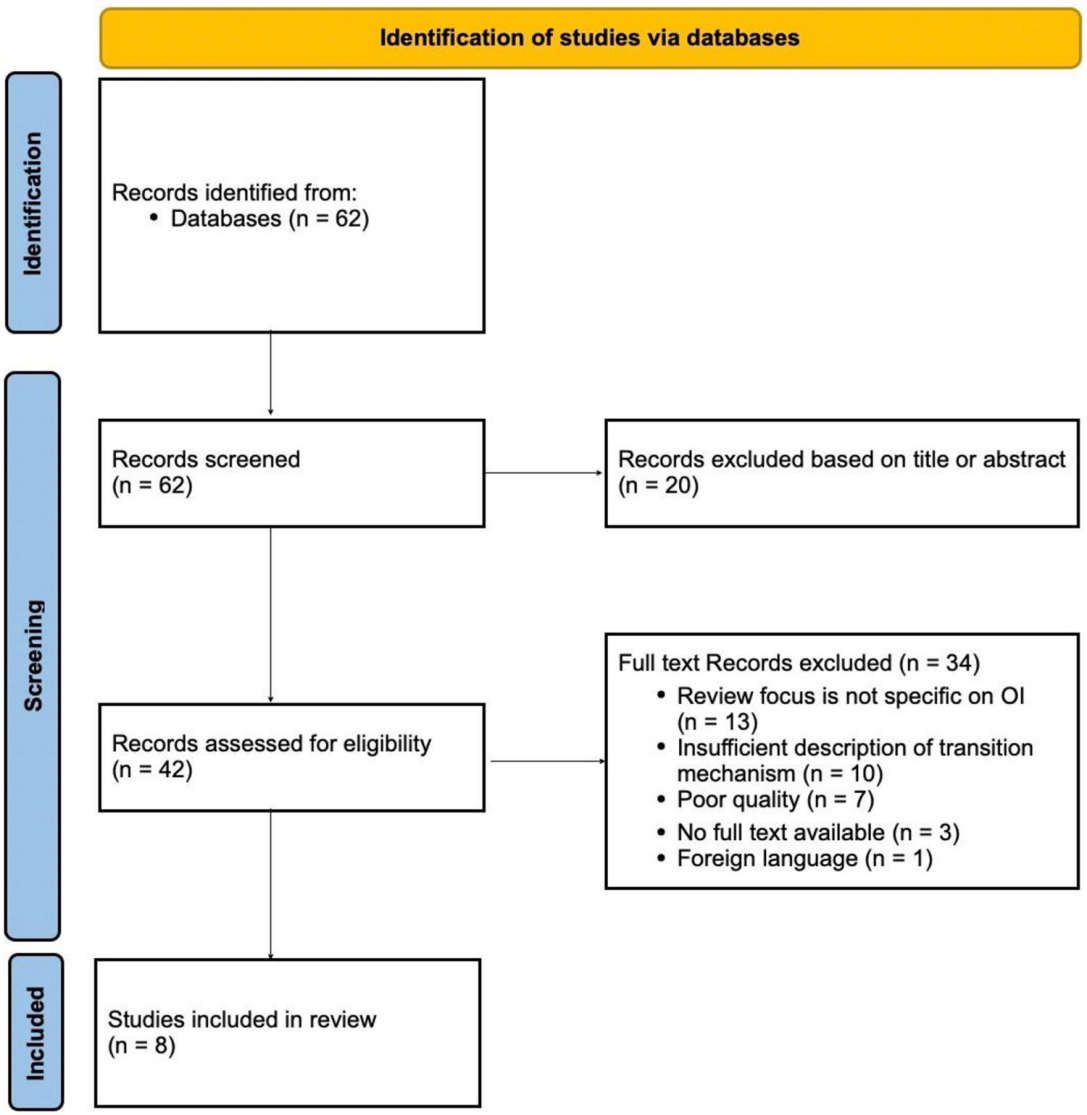
PRISMA flow diagram of the study selection process. Screening of 62 available literature records on the topic ended with the selection of 8 articles, fulfilling the screening criteria and incorporated in the review of pediatric to adult healthcare transition, applicable to OI patients

After removal of duplicates, two assessors (LC and EMWE) performed independently the selection of the articles. The initial screening included a screening of article titles, followed by a thorough review of abstracts to assess their compliance with eligibility criteria. Subsequently, studies that passed the initial screening were subjected to an exhaustive full text analysis to ensure their relevance to OI pediatric to adult care transition process (Fig. 1).

### Eligibility Criteria

The inclusion criteria used to assess the literature included: (a) OI patients; (b) adequate description of the OI pediatric to adult care transition; (c) publication in English; (d) availability of the full text, cases and case series, cohort studies, systematic and integrative reviews. The exclusion criteria used to assess the literature were: (a) absence of the description of the pediatric to adult transition in patients care.

### Quality Assessment

Two co-authors (LC and EMWE) reviewed independently the methodological quality of the full text publications using the Rayyan webtool. Study characteristics including the authors, year of publication, study design, cohort, aim and findings are presented in Table 1, 2.

**Table 1:** Articles on transition of OI patients from pediatric to adult care included in the review based on the Preferred Reporting Items for Systematic Reviews and Meta-Analyses (PRISMA) selection process

#	Authors, Year	Country of Origin	Study Design	Study Purpose	Study Cohort	Study Findings
1	Shapiro and Germain-Lee, [32]	United States	Opinion paper	To create guidelines for pediatricians, adult physicians across various medical disciplines, and families of patients planning the transition of adolescents with OI to adult care	NA	Four crucial aspects of transitioning are: Maintaining health during the transition; Preserving or enhancing functional abilities; Ensuring continuity of medical and surgical care; Restructuring psychosocial and work-related systems. Successful transition requires active communication between pediatric and adult healthcare teams, proactive involvement from the patient and their family. Patients are encouraged to become their own advocates and coordinators of care
2	Dogba et al. [23]	Canada	Qualitative approach (interviews, observation, document review)	To evaluate a transition from pediatric orthopeadic hospital to adult care for adolescents and young adults with OI using a “Strengths, Weaknesses, Opportunities, and Threats” (SWOT) approach	6 OI patients, 4 parents, and 15 health care professionals (multidisciplinary)	Individualized transition model for OI patients is necessary. It includes customized, theoretically sound programs reflecting patient preferences, aligned with organizational activities, and continually evaluated Strengths of transition models: a partnership with parents and fostering independent living and professional integration Challenges: organizational changes, discontinuation of care, and a potential conflict between the transition program and participation in research protocols
3	Carrier et al. [22]	Canada	Literature review, task force discussions	To review literature, formulate transition guidelines and develop a tool for transition	NA	The ‘‘OI Transfer Summary’’ tool provides guidelines for a smooth transition from child to adult healthcare services, creating a person-focused OI transfer tool to improve adolescents’ transition experiences
4	Jeong et al. [33]	Canada	Systematic literature review, task force discussions	To systematically review and synthesize research on the Good2Go MyHealth Passport Program; the use of portable health-related tools for care transitions; and care transitions for individuals with OI. Insights from these reviews informed the development of the MyHealth program	NA	The development of the OI MyHealth Passport enhances the resources available to the OI community, aiding in the facilitation of care transitions
5	Michalovic et al. [34]	Canada	Qualitative approach (interviews)	To investigate the self-management needs perceived by young adults with OI in order to enhance self-management and transitional care services	7 OI patients	OI patients experienced frustration from transition. Adolescents and young adults with OI need education and mentorship, while adult healthcare clinicians need preparation and support. Collaborative efforts are essential to enhance self-management and transitional care for young adults with OI
6	Shaw et al. [35]	United Kingdom	Qualitative approach (focus groups)	Investigate parents’ perceptions and experiences of their caregiving role during their child’s transition to adult services, identify their needs, and guide service improvements	3 OI patients	Parents use protective strategies, and hinder adolescent development as midlife adults. Balancing immediate health protection and long-term well-being is important. Participants sought better support and improved service organization, offering recommendations for managing risks and uncertainties in routine transitional care. Families would benefit from a more holistic approach that incorporates how young people and parents experience and understand risk, uncertainty, and vulnerability. Collaboration with parents and young people to create proactive plans to handle risks and uncertainties, making this a standard aspect of transitional care is needed
7	Rauch and Morin [31]	Canada	Systematic literature review	Review on challenges of the OI pediatric to adult-focused health care transition	NA	OI clinical impact changes with age; adults experience fewer fractures but more prominent extraskeletal symptoms. Some tools, like self-management programs, have been created. Transition processes differ across health care systems, complicating generalization. Clearer guidelines for adult follow-up and care could improve the transition from pediatric to adult health care
8	Tsimicalis et al. [36]	Canada	Qualitative approach (interviews, task force discussions)	To create the user-centered “Teens OI” tool, and evaluate its usability by assessing its efficiency and satisfaction from the viewpoints of youths with OI and their parents	13 OI patients, 4 health care professionals, 1 parent	There is a need to prioritize mental health, pain, accessibility, medical care, education, community, and parental care for youths with OI. The study developed a self-management and transitional care program, leveraging eHealth technologies to help youths manage their conditions and transition to adult care, known as “Teens OI” tool, which addresses a critical gap in care quality and aims to reduce adverse events during the transition from pediatric to adult care

**Table 2:** Articles on transition of patients with other (rare bone) disorders from pediatric to adult care included in the review based on the Preferred Reporting Items for Systematic Reviews and Meta-Analyses (PRISMA) selection process

**#**	Authors, Year	Country of Origin	Study Design	Study Purpose	Study Cohort and Disease	Study Findings
1	Grant and Pan [37]	Canada	Comparative study	Review of five transition programs for youth with chronic illnesses aligning guidelines	NA	Existing transition programs inconsistently enforce the published recommendations by the Canadian Pediatric Society and the Society of Adolescent Health and Medicine, including providing patients with a portable summary of their healthcare needs. The effectiveness and outcomes of transition programs need to be evaluated
2	Alsufyani et al. [38]	Saudi Arabia	Cross-sectional study	Evaluate the readiness of patients to be transitioned from pediatric to adult care using the Transition Readiness Assessment Questionnaire (TRAQ)	54 patients with inflammatory bowel disease (IBD)	The IBD patients experience limited guidance and unknown influential factors during transition to adult care. Older patients have higher transition readiness, with potential disparities based on income and healthcare access. Males, especially in tracking health issues, tend to score higher, while patients under 15 show lower readiness. This underscores the need for targeted interventions to improve transition preparedness
3	Currie et al. [39]	Canada	Qualitative approach (focus groups)	To investigate the experience of young people during the process of transition	9 patients with juvenile idiopathic arthritis (JIA)	A critical need for preparation, social support, mental health support, and continuity of care during the transition from pediatric to adult care was emphasized. The study highlighted the importance of effective communication with healthcare providers and post-transition support. Transition-related challenges can cause stress and affect health, underscoring the need for resources and ongoing support throughout the transition process
4	White and Cooley [40]	United states	Clinical report	To provide practical quality improvement guidance for transitioning youth and young adults into adult care, covering transition planning, transfer, and integration	NA	The clinical report includes updates on definitions, guiding principles, transition preparation, barriers, outcomes, recommended processes, and implementation strategies. It addresses special populations, education, training, payment options, and offers new recommendations on infrastructure, education, training, payment, and research
5	Ross et al. [41]	United states	Systematic literature review	To offer a concise review of research and guidelines for care transitions in general, followed by a more in-depth discussion focused specifically on bone disorders	NA	There is a need for robust infrastructure and multidisciplinary teams to support the transition of patients with metabolic bone disorders from pediatric to adult care. Evidence-based practices, addressing challenges like bone mineral density measurement standards and treatment adjustments are essential. Telehealth is a potential solution for equitable care. The gaps in limited guidelines and literature on recommendations, emphasizing the importance of continued research and strategic care progression to improve patient outcomes and quality of life
6	Scognamiglio et al. [42]	Italy	Qualitative approach (observation, focus groups)	To determine the primary priorities in transitioning of patients from pediatric to adult healthcare	30 patients with Multiple Osteochondromas, Ollier Disease, and Maffucci Syndrome,, 61 caregivers, 17 healthcare professionals, 7 healthy siblings	The collaborative framework involving patients, families, and healthcare providers, with psychological support is a top priority. The transition requires patients to self-advocate and includes key factors such as a defined care plan, involvement of a general practitioner, and the establishment of a digital health record. The study underscores the value of stakeholder participation in shaping transition protocols and provides insights for future research and protocol development

## Results

### Search Results

The literature search identified 62 publications after removal of duplicates (Fig. 1). After title screening of all 62 records, 42 articles were assessed as eligible for following abstract screening. The abstract screening resulted in 8 articles, all of which discussed transition in healthcare for OI [22, 23, 31–36]. In addition, the study separately included six recent articles extensively devoted to transition to other known chronic diseases based on a separate general search (not shown here) [37–42]. Summary of the articles included in the current review is given in Tables 1 and 2.

### Factors Ensuring Success of OI Pediatrics to Adult Care Transition

Although different transition processes exist across various healthcare systems, the reviewed literature stratifies the experiences and guidelines of OI patients’ transition from pediatrics to adult care into several generalized key topics, which create critical elements to secure the success of the well-coordinated transition process. Interestingly, all available literature clearly emphasizes the need for improved guidelines for adult follow-up and care to improve transition outcomes. Challenges such as organizational changes, care discontinuation, and conflict of transition programs with ongoing research protocols must be carefully considered.

#### High Standards of OI Health Care and Transition Requirements

Maintaining health care during transition and the continuity of medical care from a multidisciplinary team after transition is crucial [32]. Thus, a single healthcare provider overseeing the patient’s care, ensuring a comprehensive view and integrating it into sound advice and policy, is essential. This lead healthcare provider keeps all disciplines informed and engaged in the specialized OI care, which is typically provided alongside their own specialties [32]. To ensure smooth care delivery, synergistic cooperation among the various involved disciplines and a clear allocation of tasks between clinicians and nurses is vital. The transition for individuals with OI is a complex process requiring a multidisciplinary approach to ensure high quality of care [31, 34]. Studies suggest that a structured, coordinated transition is crucial to avoid the fragmentation of services, which often occurs in adult care settings where the specialized networks of pediatric institutions may be lacking [31, 34]. The systemic nature of OI necessitates ongoing care from specialists like orthopedic surgeons, endocrinologists, audiologists, pulmonologists, cardiologists, ENT doctors, and rehabilitation professionals. However, adult care settings often fail to provide the same level of coordination, leading to disjointed treatments from various providers without a central coordinating role [22, 23].

The time taken for the consultation to transition of a patient from pediatric to adult care, undertaken by diverse medical specialists, is crucial [32]. Ensuring continuous, high-quality care fulfills the patient’s well-being and sense of security, particularly during the transition to adulthood [23]. Healthcare providers should address patients’ fears of pain and fractures, reassuring them of the “new” adult OI care team’s extensive experience with handling of OI patients, who need special management, fracture and pain treatment [35, 36]. This process demands a high level of expertise, and patience to earn the trust of an adapting OI patient. Recognizing fatigue during consultations is also vital for maintaining patient engagement in OI care. It is recommended that OI care can be provided in hospitals with specialized expertise, where the necessary organization and specialist knowledge are available. Ideally, these OI expertise hospitals should collaborate effectively with peripheral hospitals near the patient, sharing knowledge and remaining available for questions, with tailor-made check-ups. Providing an early and thorough explanation of the potentially different structure and organization of adult care facilities is important.

#### Individualized Approach of Transition Models

Variability of healthcare systems, as well as variability of OI representation necessitates customized programs reflecting patient preferences. Personalized transition plans that take into account the unique needs and preferences of each patient are key to smooth transition [23]. These individual transition plans benefit from continuous evaluation and ongoing adjustments to ensure that they remain effective. Moreover, such customized individual plans are flexible enough to align with organizational activities, as transition programs have to be integrated within the healthcare center.

#### Shift in Prioritizing of Key Areas for OI Youth/Young Adults Well-Being

Qualitative research of transition experiences, as well as previous questionnaires of adults with OI underline the shift of priorities in OI patient well-being and care after pediatric age, underlining changes in clinical impact of OI with aging [31, 43]. Focus from fractures and skeletal concerns changes to extraskeletal challenges, with highest impact on quality of everyday life affected by pain and fatigue, which is shared by (young) adult and adolescent OI patients [15]. According to adolescents, psychosocial components of the well-being also gain critical importance (e.g. accessibility, education, mental health, parental care and independence) [36]. The programs should address the broader impact of transition on young patients’ social lives and work readiness, ensuring adequate preparation for adult responsibilities.

#### Communication and Advocacy

A proactive patient and family involvement are an inherent component of the transition process. Young patients should be encouraged to take an active role in the transition process, promoting self-advocacy and coordination of care. At this moment partnership with parents is extremely important for a successful transition and fostering of a supportive environment [35]. Parents should not be fully excluded during and after the transition. However, protective strategies used by parents and their impact on OI youth undergoing transition, should be made clear with special attention to holistic approaches, which include understanding the experiences and perceptions of risk and vulnerability by both young people and their parents. This must be thoroughly discussed with the coordinating medical specialist, patient, and parents. Long-term parental assistance for this may be required.

Understanding frustration within all parties participating in the transition is equally important [34]. The young patient experiences challenges and frustrations related to the “unknown” status during the transition, and relies on mentorship and education to gain full independence. In addition, clinicians should be prepared and supported in managing the care of transitioning patients, who might need more guidance.

#### Transition Tools and Resources for OI Youth

A number of transition tools were created by Canadian researchers to support OI transition.

(a) OI Transfer Summary Tool [22]: Provides a guidelines for a smooth transition, focusing on personalized care (available online and as pdf). It is an intriguing concept designed to ease the transition from pediatric to adult care. In hospitals with established electronic file systems, data can be seamlessly shared among healthcare providers, facilitating a straightforward transfer tool. However, in locations where such systems are not yet common, transferring the care history and various elements of the current pediatric care path becomes crucial.

(b) OI MyHealth Passport [33]: The tool enhances available organizational resources, aiding in the facilitation of care transitions, while ensuring gathering of patients’ data via self-management;

(c) Teens OI [36]: is an eHealth-based program to help youths manage their conditions and transition to adult care, empowering self-management for OI youth.

### Transition to Adult Care Lessons from Other Chronic Diseases

**Fig. 2:**
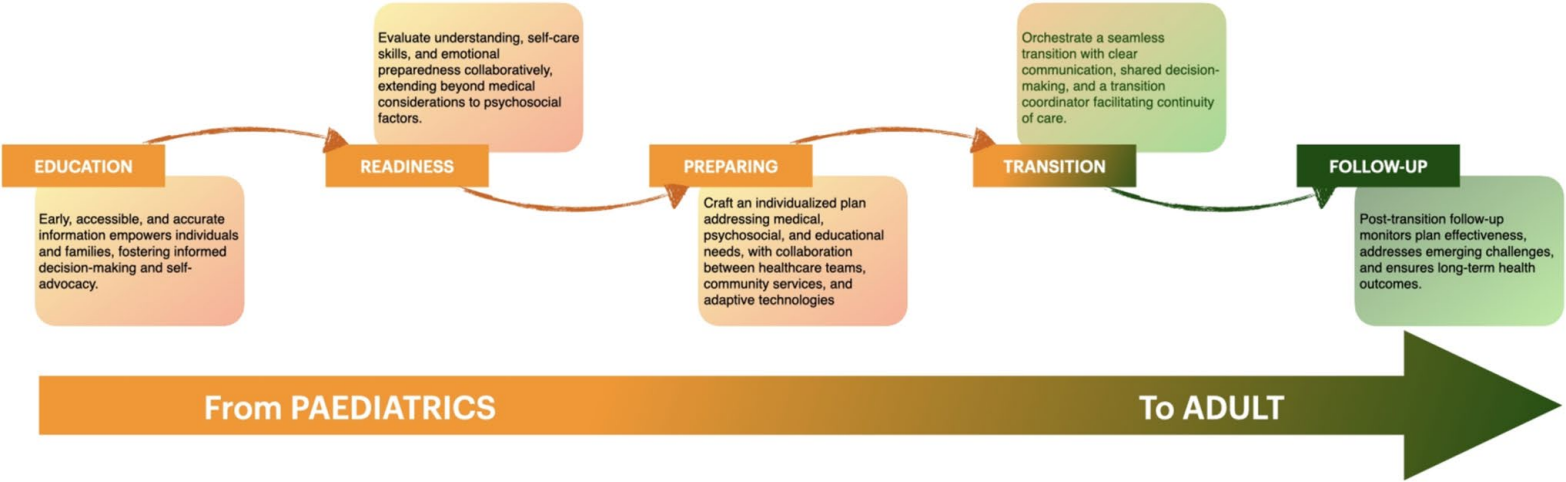
Framework scheme encompassing five key steps (e.g. Education, Readiness, Preparing, Transition, Follow-up) of transition from pediatric to adult health OI care

As more research has been conducted on transition in other chronic diseases, we reviewed the latest literature to determine if there are any important practices to consider in the context of transitioning within OI. From this literature the following structured common key preparatory steps emerged, aimed at preparing the adolescent for adult care: 1. Education, 2. Readiness, 3. Preparing, 4. Transition and 5 [35, 36, 43], Follow-up of the transition [38, 39, 44] (Fig. 2). In addition, peer support for the young adult also appeared to be of great importance outside the transition of care, as well as a responsible professional (doctor) who guides and monitors the transition. The latter may be the doctor himself or a representative of a healthcare organization. The medical knowledge transfer and the establishment/presence of a medical competent multidisciplinary team for adult care (care pathways) is also indispensable.

Below we provide some examples.

Education is necessary to be ready for the transition. This has been highlighted in several articles on type 1 diabetes, juvenile arthritis and irritable bowel syndrome [38, 39, 44]. As an example of the latter, the readiness of Saudi adolescents with inflammatory bowel disease to transition from pediatric to adult care was assessed in a cross sectional study using the Transition Readiness Assessment Questionnaire (TRAQ). It turned out that the child’s level of education was the only independent variable that correlated with higher total scores. Patients over 15 years old had higher overall scores than younger patients [38].

Because the transition plays a major role in young people with type 1 diabetes (T1D), the American Academy of Pediatrics, American Academy of Family Physicians, and American College of Physicians jointly published the 2018 Clinical Report on the Guiding Principles of Healthcare Transition (HCT) [40]. This clinical report outlines six core elements of HCT in a structured process, which have been tested through partnership building using quality improvement methods. These have led to packages of the six core elements, available with resources and implementation guides. The six core elements include guide/policy (what to expect), tracking and monitoring (to ensure the adult is receiving the six core elements), readiness (of the adolescent), transition planning, transfer of care, transition confirmation (follow-up and feedback to the pediatric practice). However, they specifically target T1D [44].

In Juvenile arthritis (JIA) a patient-led study provided insight into the experience from the perspective of young people with JIA. They used a patient and Community Engaged Research (PaCER) approach and three focus groups, which showed the importance of readiness for the transfer of care, in developing self-advocacy skills, continuity of care (changing relationships, new responsibilities), need for support (social and mental health, beyond the transfer of care). Peer support was experienced as a very important overarching factor [39].

## Discussion

In 2002, the American Academy of Pediatrics (AAP) together with the American Academy of Family Physicians (AAFP) and the American College of Physicians-American Society of Internal Medicine (ACP-ASIM) stressed the importance of transition by releasing a first consensus statement [45]. This outlines six steps to ensure that young adolescents with special health care needs receive the required support to transition to adult-oriented medical care. These steps included 1. Identification of a health care professional equipped to address the unique challenges of transition; 2. Determination of the core knowledge and skills necessary to provide developmental phase-appropriate health care transition services to adolescents with the aim its implementation in training and certification requirements for physicians; 3. Preparation and maintenance of an up-to-date medical summary that is portable and accessible; 4. Development of a healthcare transition plan based on collaboration together with the adolescent and the family before the age of 14. This plan must include the provided services, health care provider and financing. Annual plan review and revision must accompany every transfer of care; 5. Application of uniform primary and preventive care guidelines to adolescents and young adults; recognition of the special health care needs which may require allocation of more resources and services; 6. Provision of affordable and continuous health insurance coverage for all adolescents with special health care needs throughout adolescence and adulthood, including appropriate reimbursement for a) care transition planning for special health care needs, and b) care coordination for complex medical conditions [46]. Based on these points, transition guidelines have emerged for chronic conditions, but to much less extent for many rare conditions. Their inherent complexity poses unique issues and concerns which, in combination with variable healthcare structures, has complicated their implementation across countries. Thus, even though the generation of an overarching manual is necessary it can also be a challenge.

As a follow-up to the published consensus, the National Alliance to Advance Adolescent Health (2023) has developed the Got Transition® program in collaboration with the American Academy of Pediatrics, the American Academy of Family Physicians, and the American College of Physicians [45]. This offers several condition-specific tools including transition readiness assessment**,** medical record and self-assessment for aiding clinicians with optimizing the transition process of young adults with chronic health conditions, which has been expanded for several specialisms, albeit not yet for rare bone diseases. In addition, the role of parental guidance in transition has increasingly gained attention as an important cornerstone for tailor-made transition guidance [45, 47]. Since this is a gradual process of the parents introducing their maturing child to transition, parents may need guidance with the transfer of responsibility, support, conflict management and trust building from early child adolescence. This can alleviate parents’ anxieties and improve the process [48] for which reason it is advised to include this early in the transition plan.

The literature on transition in OI is still scarce, but slowly gaining more attention as a decisive factor for quality of life. A first few general guidelines recently emerged that have been developed based on the patients’ experience and the investigation of needs in adolescents and adults [25, 49]. The eight articles about transition in OI identified in this study address different aspects of transition in healthcare. Although they all involve small patient groups and/or small focus groups, it is encouraging that these initiatives are being undertaken for a disease as diverse, complex and rare as OI. The identified studies emphasize the importance of a well-organized transition process. Michalovic et al. [31] exposed the emotional challenges which are experienced during transition [34]. In line with this, Shapiro et al. described the importance of maintaining health care and communication [32]. Scognamiglio et al. also underlined the need for a collaborative framework involving the patients, families and healthcare providers, and stressed the importance of addressing the psychological aspects [42]. The comprehensive model of Dogba et al. promoted partnership with the parents facilitating independent living and professional integration [23]. Carrier et al. provided a template for an OI transfer tool [22], whereas Jeong et al. suggested that the eHealth passport can be utilized as a transfer tool [33]. New initiatives on managing online OI transition are in development, e.g. a tool by Tsimicalis et al., called “Teens OI” [36], which with appropriate level of promotion might serve as a standard for the transition OI care for some of the national healthcare systems.

In Europe, expertise centers must meet the European Reference Networks (ERNs) requirements to guarantee protocols with OI-dedicated care paths and transitions, involving a specific number and types of disciplines. However, different countries offer different transition methods, which should be taken into consideration. In certain European countries, pediatric care is well-organized, but the transfer to adolescent care is not always clearly structured. In some cases, the pediatrician remains the primary caregiver and collaborates with adult disciplines. Additionally, an orthopedic surgeon may oversee the care for children and continue after their transition, integrating the various aspects of care in older age. In other countries, the transfer from child to adult care may occur through a single joint consultation during the transition or gradually over years of combined consultations. Sometimes, arranging care for adults is challenging due to insufficient attention from adult care specialists, given the rarity of OI. Consequently, the lack of a transition program in a hospital may affect eligibility for ERN BOND membership, despite the high expertise level of pediatric care.

The need in OI for robust preparation, social support, and continuous care during the shift from pediatric to adult services is also critical in cases of other diseases. Effective communication and post-transition support to reduce stress and enhance health outcomes are highly used among patients with chronic conditions. Based on experience of other disorder communities, multidisciplinary teams, telehealth solutions, and evidence-based practices are vital for improving patient transitions, with stakeholder collaboration and ongoing research being pivotal in shaping future protocols. Equally important is the rigorous evaluation of patient satisfaction with transition programs which can facilitate their critical revision and continuous improvement. The use of patient-reported outcomes (PROMS) can help to assess the effectiveness of the course and clinical practices within the transition plan [31, 34, 49]. Although standards and guidelines of transition to adult care in the OI community are still being developed, prosperous practices from other fields can be implemented in OI transition.

### Recommendations for an Adapted Structured Five-Step OI Transition Approach

A structured framework, encompassing five key steps can be evolved from the recommendations and experiences of a successful transition: Education, Readiness, Preparation, Transition, and Follow-up (Fig. 2).

#### Education

Comprehensive education is fundamental in empowering individuals with OI and their families. From an early age, it is vital to provide accessible and accurate information on OI, its complications, and available resources. Healthcare professionals must engage in ongoing education to ensure that patients and their families fully understand their healthcare needs and potential complications. This education fosters crucially important informed decision-making and self-advocacy, which is essential for adult independency.

#### Readiness

As individuals approach the transition phase, assessing their readiness for adult care is crucial. Readiness includes understanding the condition, developing self-care skills, and being emotionally prepared. Healthcare providers should work closely with patients to identify their strengths and areas where additional support may be needed. Readiness assessments must also consider psychosocial factors and the development of a support network and mentorship. Influence of age, and personal traits of the patient on readiness should be taken into account.

#### Preparation

Developing an individualized transition plan is vital for preparing the patient for adult care. This plan should encompass medical, psychosocial, and educational needs. Collaboration between pediatric and adult healthcare teams is crucial, along with integrating community resources, vocational training, and adaptive technologies. This ensures that patients are well-prepared for their new phase of care.

#### Transition

The transition itself should be carefully orchestrated, involving clear communication and shared decision-making between the patient, family, and healthcare providers. A transition coordinator can play a critical role in ensuring the smooth transfer of medical records, treatment plans, and ongoing care. Encouraging active participation from individuals with OI in their healthcare decisions during this phase fosters autonomy and confidence.

#### Follow-Up

Post-transition follow-up is necessary to monitor the effectiveness of the transition plan and address any emerging challenges. Regular check-ups, ongoing education, and adjustments to the transition plan ensure long-term success. Building a strong rapport between the patient, (family), and healthcare providers creates a supportive environment that enhances health outcomes.

This five-step approach prioritizes informed decision-making, self-advocacy, and collaboration, allowing individuals with OI to navigate adult care with resilience and confidence.

## Conclusions

Our study underscores the importance of structured transition programs for individuals with OI. Currently, only a few such programs exist, which may be often underutilized or poorly implemented. Successful transition programs for other chronic conditions generally include formalized plans that start during adolescence, patient education on disease management, and a coordinated transfer to adult services. Our findings suggest that applying a similar model to OI could reduce healthcare fragmentation and improve patient confidence.

The transition from pediatric to adult care for individuals with OI is a multifaceted process that demands a comprehensive and patient-centered approach. By applying a structured framework that includes education, readiness assessments, preparation, and follow-up, healthcare providers can ensure a seamless transition, benefiting patients, and their families and caretakers. Collaborative efforts among cardiologists, ophthalmologists, orthopedic specialists, and medical geneticists are essential for addressing the complex needs of OI patients. This approach not only improves patient outcomes but also contributes to the ongoing research efforts aimed at optimizing care for those living with OI. Further experimental studies are planned to assess the feasibility of this stepwise transition system for OI patients.

## Data Availability

All relevant data are presented in the current study.
